# Microbiological Profile and Bioactive Properties of Insect Powders Used in Food and Feed Formulations

**DOI:** 10.3390/foods8090400

**Published:** 2019-09-09

**Authors:** Concetta Maria Messina, Raimondo Gaglio, Maria Morghese, Marco Tolone, Rosaria Arena, Giancarlo Moschetti, Andrea Santulli, Nicola Francesca, Luca Settanni

**Affiliations:** 1 Laboratory of Marine biochemistry and ecotoxicology, Department of Earth and Marine Science DISTEM, University of Palermo, Via G. Barlotta 4, 91100 Trapani, Italy; 2 Department of Agricultural, Food and Forestry Science, University of Palermo, Viale delle Scienze 4, 90128 Palermo, Italy

**Keywords:** Alcalase, insect powders, *Acheta domesticus*, *Tenebrio molitor*, *Enterococcus*, antioxidant activity

## Abstract

Microbiological, nutritional and bioactive properties of edible powders obtained from *Acheta domesticus* (house cricket) and *Tenebrio molitor* (mealworm) were investigated. Except for the enterobacteria, viable bacteria were at a higher concentration in mealworm flour. The diversity evaluation carried out using MiSeq Illumina that mainly identified *Citrobacter* and *Enterobacteriaceae* in mealworm powder and members of the *Porphyromonadaceae* family in house cricket powder. Enterococci were identified and characterized for their safety characteristics in terms of the absence of antibiotic resistance and virulence. Both powders represent a good source of proteins and lipids. The fatty acid profile of mealworm powder was characterized by the predominance of the monounsaturated fatty acids and house cricket powder by saturated fatty acids. The enzymatic hydrolysis produced the best results in terms of percentage of degree of hydrolysis with the enzyme Alcalase, and these data were confirmed by SDS-PAGE electrophoresis. Furthermore, the results showed that the protein hydrolysate of these powders produces a significant antioxidant power.

## 1. Introduction

Insect consumption occurs almost worldwide, and this practice would represent a potential solution to food shortages and famine [1,2]. The nutritional relevance of insects is mostly represented by their high digestible protein content [3]. Compared to conventional livestock, their breeding systems are characterized by fewer environmental issues, including lower water consumption [4], greenhouse gases and ammonia generation [5].

Insect consumption in Western countries is still limited [6,7]. First of all, because of the unpleasant perception that the majority of consumers have towards such foods, which are not considered as conventional [8]. The consumption of edible insects is also hampered by regulations regarding hygiene and safety issues. Furthermore, religious concerns should also be considered in future. In areas of Asia, Africa and South America, where insects are eaten daily, they are commonly collected from natural environments [9]. Thus, the microbiological load implications of these foods might be relevant. Durst et al. [10] reported some cases of botulism and other foodborne illnesses due to the consumption of insects stored in Africa. The major risks derive from the ingestion of the gastrointestinal tract of the insects [11]. However, several commercial insect farms are keeping the growth of edible insects under controlled hygiene conditions.

In Western countries, insects are often consumed as flour added to some traditional food ingredients in several formulations. Insect powders were mixed with maize flour to produce tortillas [12] and emulsion sausages [13]. Commonly used species is the mealworm *Tenebrio molitor* (*T. molitor*) [14], but house cricket *Acheta domesticus* (*A. domesticus*) also showed a high potential for the enrichment of food products, including fermented ones [11]. *T. molitor* and *A. domesticus* and their powders have been studied for microbiological aspects showing a consistent presence of *Enterobacteriaceae* family [11,14,15,16,17], techno-functionality, chemical and nutritional composition showing high quantity of crude proteins, micronutrients, B-group vitamins and low crude fat [14,18,19].

Currently, there is a growing interest in the applications of food proteins and peptides in the form of functional foods or nutraceuticals as alternatives ingredients to conventional treatments. Enzymatic modification of proteins is useful to improve functionality [20]. Some peptides obtained from dietary proteins using enzymatic hydrolysis have been demonstrated to be antioxidant, antimicrobial, antidiabetic, antihypertensive, antithrombotic and immunomodulating [21,22].

Edible insects are viable sources of bioactive peptides owing to their high protein content and sustainable production [20,23]. A multidisciplinary approach consisting of chemical/nutritional, biochemical and microbiological investigations has been applied to characterize mealworm and house cricket powders.

## 2. Materials and Methods

### 2.1. Raw Materials and Microbiological Analyses

The powders analyzed were prepared from *T. molitor* and *A. domesticus* insects (two samples for each species) and were provided by Kreca Ento-Food (Harderwijk, Gheldria, The Netherlands). As reported in labels, both insect powders are important sources of proteins, since they contain all the nine essential amino acids. Furthermore, both powders have a high digestibility, possess a low carbohydrate profile and are free of preservatives, antibiotics, hormones and pesticides. The powders were kept under refrigeration in the dark as suggested by the supplier. To evaluate the changes of the chemical/nutritional, biochemical and microbiological characteristics of mealworm and cricket powder during storage, the powders were analyzed after 12 months of refrigerated storage (4 °C).

The insect powders were subjected to decimal serial dilution in Ringer’s solution (Sigma-Aldrich, Milan, Italy). The first dilution was obtained using a Stomacher (BagMixer^®^ 400, Interscience, Saint Nom la Bretèche, Yvelines, France) at the highest speed (260 rpm) for 2 min. Cell suspensions were measured using plate count for the enumeration of the following microbial groups: total mesophilic microorganisms (TMM) on plate count agar (PCA) incubated aerobically at 30 °C for 72 h; mesophilic lactic acid bacteria (LAB) rods and cocci on de Man-Rogosa-Sharpe (MRS) and M17 agar, incubated anaerobically at 30 °C for 48 h; enterococci on kanamycin esculin azide (KAA) agar incubated aerobically at 37 °C for 24 h; members of the *Enterobacteriaceae* family on violet red bile glucose agar (VRGBA) incubated aerobically at 37 °C for 24 h; coagulase-positive staphylococci (CPS) on Baird-Parker (BP) agar supplemented with rabbit plasma fibrinogen (RPF), incubated aerobically at 37 °C for 48 h; pseudomonads on *Pseudomonas* agar base (PAB) supplemented with cephaloridine sodium fusidate cetrimide (CFC), incubated aerobically at 25 °C for 48 h; yeasts and moulds on malt agar (MA) with chloramphenicol (0.1 g/L), incubated aerobically for 48 h and 7 days, respectively, at 28 °C; spore-forming aerobic bacteria were investigated after heating of cell suspensions at 85 °C for 15 min and then spread plated on nutrient agar (NA) before aerobic incubation at 32 °C for 48 h. Anaerobiosis occurred in hermetically sealed jars with the AnaeroGen AN25 system (Oxoid, Milan, Italy). All media were purchased from Oxoid. Microbiological counts were carried out in triplicates.

### 2.2. V3-V4 Amplification and Illumina Data Analysis

To maximize the effective length of the MiSeq’s 300PE sequencing reads, the region encompassing the V3 and V4 hypervariable regions of the 16S rRNA gene (approximately 469 bp) was targeted for sequencing. Genomic DNA was extracted from insect powder samples using a QIAamp DNA Mini Kit (Qiagen, Hilden, Düsseldorf, Germany) and diluted to 5 ng/μl in 10 mM Tris pH 8.5 as indicated using the Illumina protocol 16S Metagenomic Sequencing Library Preparation, 15044223 Rev. B. Briefly, to amplify and sequence the V3-V4 hypervariable region of the 16S rRNA gene, primers were designed that had overhang adapter sequences that must be appended to the primer pair sequences for compatibility using the Illumina index (San Diego, CA, USA) and sequencing adapters. The libraries were sequenced using the MiSeq Reagent Kit v3, 600 Cycles Sequencing kit (MS-102-3003) on the MiSeq System (Illumina).

Sequences obtained from Illumina Sequencing were processed using the QIIME2 software package version 2018.4 [24]. The reads were assigned to each sample according to the unique index; pairs of reads from the original DNA fragments were first merged using an import tool implemented in QIIME2. Quality check and trimming were done to trim sequences where the Phred quality score was < 20 using the DADA2 a R packages [25] wrapped in QIIME2. The Phred quality score is a measure of the quality of the identification of the nucleobases generated by automated DNA sequencing. Moreover, to remove chimeras from the Illumina sequenced FASTQ files the “consensus” method implemented in DADA2 was used. For taxa comparisons, we used the QIIME2 q2-feature-classifier plugin and the Naïve Bayes classifier that was trained on the Greengenes 13.8 database with a 99% Operational Taxonomic Units (OTUs) full-length sequences. QIIME2 taxa barplot command and ggplot2 were used for visualization of the taxonomic composition of the samples. Alpha diversity analysis was done with the q2-diversity plugin in QIIME2. In particular, Chao1 [26] metric that is a nonparametric abundance-based estimator of species richness and observed OTU were used to study diversity within each sample. Finally, to compare the relative abundance of microbial communities between the two samples, a Kruskal-Wallis test was done [27].

### 2.3. Phenotypic and Genotypic Characterization of LAB

Some colonies of presumptive LAB (Gram-positive, determined using the Gregersen KOH method [28], and catalase negative, determined by addition of fresh colonies from the agar media to 5%, *w/v*, H_2_O_2_) from the highest plated dilutions of the microbial cells on MRS, M17 and KAA agar were collected for all different morphologies recognized considering color, shape, edge, and surface (smooth or jagged). Gram-positive and catalase-negative cultures were purified through successive sub-culturing in the same media used for plate counts. All cultures were characterized for their cell morphology determined using an optical microscope at 100 × (Zeiss, Oberkochen, Stuttgart, Germany), growth at 15 and 45 °C, metabolism type, testing the ability to produce CO_2_ from glucose and growth in the presence of a mixture of pentose carbohydrates (xylose, arabinose and ribose; 8 g/L each) in place of glucose [29]. The coccus-shaped isolates were finally tested for their growth at pH 9.2 and in the presence of 6.5% (*w/v*) NaCl.

Genomic DNA from the PCR assay was prepared using the InstaGene Matrix kit (Bio-Rad, Hercules, CA, USA) as described by the manufacturer. Cells were harvested from insect flour isolated cultures grown overnight in MRS or M17 broths at 30 °C, and genomic DNAs were extracted using the Instagene Matrix kit (Bio-Rad), as described by the manufacturer. Crude cell extracts were used as templates for the polymerase chain reaction (PCR).

Strain differentiation was done using random amplification of polymorphic DNA (RAPD)-PCR analysis using the single primers M13, AB111, and AB106 as previously described by Gaglio et al. [30] using a T1 Thermocycler (Biometra, Göttingen, Germany) to generate amplicons. The software package Gelcompare II Version 6.5 (Applied Maths, Sint-Martens-Latem, East Flanders, Belgium) was used to analyze the LAB profiles.

Gene sequencing of 16S rRNA was using as reported by Weisburg et al. (1991) with the primers rD1 (5′-AAGGAGGTGATCCAGCC-3′) and fD1 (5′-AGAGTTTGATCCTGGCTCAG-3′) for LAB identification at the species level. DNA fragments of about 1600 bp were purified and sequenced at Eurofins Genomics (Milan, Italy). The sequences obtained were compared with those available in the EzTaxon-e database (http://eztaxon-e.ezbiocloud.net/) with the sequences of the type strains only and the GenBank/EMBL/DDBJ (http://www.ncbi.nlm.nih.gov). The unequivocal identification of the *Enterococcus faecium* was further verified using the sodA gene-based multiplex PCR described by Jackson et al. [31] using the primers FM1 (5′-GAAAAAACAATAGAAGAATTAT-3′) and FM2 (5′-TGCTTTTTTGAATTCTTCTTTA-3′).

PCR mixture (22.5 μL total volume) included 20 μL of master mix and 2.5 μL of whole-cell template. The PCR program applied for all primers comprised 30 cycles of denaturation for 4 min at 95 °C, annealing for 1 min at 55 °C, and elongation for 1 min at 72 °C. Amplification was followed by a final extension at 72 °C for 7 min. The amplifications were performed using a T1 Thermocycler.

(Biometra) and the amplicons were separated by electrophoresis on a 2% (*w/v*) agarose gel (Gibco BRL, Cergy Pontoise, Val-d’Oise, France), stained with SYBR^®^ Safe DNA gel stain (Molecular Probes, Eugene, OR, USA), and subsequently visualized by UV transillumination.

### 2.4. Safety Aspects of Dominant Insect Powder LAB

The antimicrobial susceptibility of enterococci was evaluated through the standard disk diffusion method of Kirbye-Bauer according to the Clinical and Laboratory Standards Institute guidelines [32] on Mueller Hinton Agar (Oxoid) incubated at 37 °C for 18 h. The following antimicrobials were tested: penicillin—10 units, ampicillin—10 μg, vancomycin—30 μg, erythromycin—15 μg, tetracycline—30 μg, ciprofloxacin—5 μg, levofloxacin—5 μg, chloramphenicol—30 μg, quinupristin-dalfopristin—15 μg, linezolid—30 μg, high-level gentamicin—120 μg and high-level streptomycin—300 μg. All antimicrobial compounds are commonly used for the treatment of human and animal infections. *Enterococcus faecalis* ATCC 29212 was used as the quality control strain for performing antimicrobial testing. All antimicrobial compounds were purchased from Oxoid.

The phenotypic assay of gelatinase production by *Enterococcus* strains was done on a plate containing gelatin agar as described by [33]. The gelatinase production was classified as positive when a clear zone of hydrolysis was detected around the colonies. The production of haemolytic activity was determined by streaking the bacterial cultures onto Columbia blood agar supplemented with 5% (*v*/*v*) horse blood (Becton Dickinson, Franklin Lakes, NJ, USA). Plates were incubated at 37 °C for 24–48 h with anaerobic conditions, after which the plates were examined for haemolysis. The hemolytic reactions were classified as total or β-hemolysis (clear zone of hydrolysis around the colonies), partial or α-hemolysis (green halo around the colonies) and absent or γ-hemolysis.

### 2.5. Proximate Composition

The proximate composition was measured as follows: moisture and ash content using the AOAC method [34], total nitrogen using the Kjeldahl method [35]; crude protein (P) and chitin (Q) content were determined applying the following equation used by Díaz-Rojas et al. [36]:

P = ((Nt × Cq + K − 100) × Cp)/(Cq − Cp)(1)

Q = ((Nt × Cp + K − 100) × Cq)/(Cp − Cq)(2)

where Nt was the total nitrogen content. K was the sum of total lipid, moisture and ash. Cp and Cq were conversion coefficients that relate the mass fraction of nitrogen with protein and chitin. The protein content of different insect species in the literature is mainly based on nitrogen content using the nitrogen to protein conversion factor (Cp) of 6.25 [37,38] while the value of Cq is 14.5 [36].

The total fatty acid (FA) methyl esters were determined from the total lipid [37] of insect powders according to Lepage and Roy [38] and analyzed using the conditions described by Messina et al. [39] employing a Perkin Elmer (Waltham, MA, USA) autosystem XL instrument equipped using a silica capillary column (30 m × 0.32 mm, d_f_ 0.25 μm, Omegawax 320, Supelco, Bellefonte, PA, USA). Individual FAME were measured by comparison of known standards (mix of PUFA 1, PUFA 2 and PUFA 3 mixed oil, Supelco).

Caloric content was measured as total energy content (kcal/100 g) using an isoperibolic oxygen bomb calorimeter (model 6200, Parr Instrument Co., Moline, IL, USA).

### 2.6. Enzymatic hydrolysis

The samples were subjected to enzymatic hydrolysis in distilled water (1:1 *w/v*), using three different proteases (peptidases) (Protamex, Flavourzyme and Alcalase, Sigma-Aldrich). The hydrolysis reaction was performed according to Messina et al. [40] at 60 °C keeping the pH at 8.0 with the addition of NaOH 5M. These conditions are optimal for enzymatic activity [40]. The degree of hydrolysis (DH%) of each enzyme was determined directly every 15 min for 195 min, and applying the equation used by Dumay et al. [41]. The enzymatic activity was stopped at 90 °C for 5 min and the samples were centrifuged at 7142 g force for 15 min at 4 °C. The supernatants were lyophilized for further determinations and stored at 4 °C [42].

### 2.7. Sodium Dodecyl Sulphate–Polyacrylamide Gel Electrophoresis (SDS-PAGE)

The hydrolysates were separated using SDS-PAGE (SDS-PAGE, Bio-Rad). The concentration of total proteins in all samples (powder and hydrolysates) done using the Lowry et al. [43] method, using BSA as the standard assuming it was 100% pure. Aliquots of 100 µg of protein, diluted with Laemmli buffer (1970) (Sigma-Aldrich) and denaturated for 5 min at 90 °C, were loaded on a gradient polyacrylamide minigel (4–15%) (Bio-Rad) and subjected to electrophoresis at 20 mA for about 2 h. A mix of standards proteins, having relative molecular mass varying between 250 and 14 kDa (Bio-Rad) was run simultaneously into the gel. After the electrophoretic run, the gel was stained with a reagent which uses the reference protocol of staining with Coomassie Blue (GelCode Blue Stain Reagent, Pierce, Rockford, IL, USA). The image of the gel was acquired and elaborated using the software Image Lab 4.1 (Bio-Rad).

### 2.8. DPPH Radical Scavenging Activity

The total antioxidant power of the hydrolysates obtained at the end of the enzymatic processes for both insect powders was measured using the DPPH assay [44]. DPPH (1.1-diphenyl-2-picryhydrazyl, Sigma-Aldrich) is a stable free radical widely used in the detection of scavenging activity of hydrolysates for screening antioxidant compounds. Different aliquots of the sample were taken and the volume was made to 1.0 mL with ethanol. The reaction was started by the addition of 1.0 mL of 200 µM DPPH solution in 96% ethanol. The reaction mixture was kept at ambient temperature (25 °C) for 30 min and the absorbance was measured at 517 nm. Gallic acid (Sigma-Aldrich) was used as a positive control. The scavenging activity was determined using the following equation by Manuguerra et al. [45].

Scavenging activity (%) = [1 − (Absorbance sample/Absorbance control)] × 100(3)

### 2.9. Statistical Analyses

Microbiological data were subjected to one-way analysis of variance (ANOVA). Pair comparison of treatment means was done using Duncan procedure at *p* < 0.05. Differences between *T. molitor* and *A. domesticus* powders were evaluated using the generalized linear model (GLM) procedure. The linear dose-dependent relationship between the scavenging properties of the DPPH radical and the various concentrations tested has been tested through the linear regression test. The statistical analysis was done with Statistical Analysis System 9.2 software (SAS Institute, Cary, NC, USA).

## 3. Results and Discussion

### 3.1. Microbial Loads of Insect Flours

**Figure 1:**
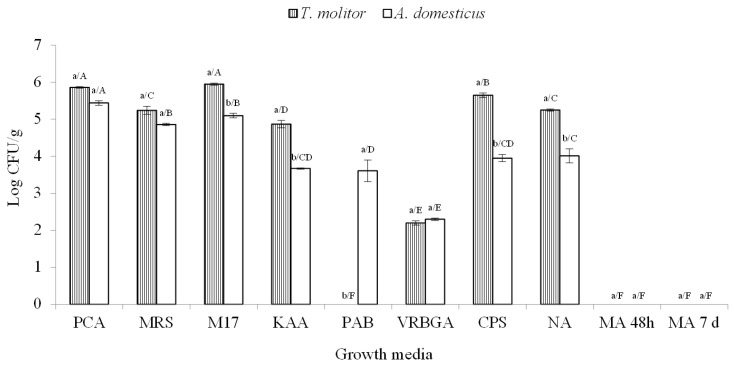
Microbial loads (Log CFU/g) of insect powders. Abbreviation: PCA, plate count agar for detection of total mesophilic microorganism; MRS, de Man-Rogosa-Sharpe agar for detection of mesophilic rod LAB; M17, medium 17 agar for detection of mesophilic coccus LAB; KAA, kanamycin esculin azide agar for detection of enterococci; PAB, *Pseudomonas* agar base for detection of pseudomonads; VRBGA, violet red bile glucose agar for detection of *Enterobacteriaceae*; CPS, coagulase-positive staphylococci; NA, nutrient agar for detection of spore-forming aerobic bacteria; MA, malt agar for detection of yeasts and moulds incubated for 48 h and 7 days, respectively. Results indicate mean values and standard deviation of three plate counts. Different lowercase letters indicate significant differences on microbial concentrations performed according to Duncan test between insect powders for *p* < 0.05 while different uppercase letters indicate significant differences on microbial concentrations between different growth media for *p* < 0.05.

Insect powders have been recently investigated for their microbiological/safety aspects by different research groups, but so far, very little is known about their characteristics at the expiry date. The microbiological investigation of *T. molitor* and *A. domesticus* powders in this work was carried out after 12 months of storage. Mealworm powder hosted 7 microbial populations, while house cricket powder was characterized by a higher microbial diversity, since the *Pseudomonas* group was also detected, forming a total of 8 microbial populations (Figure 1).

Statistically significant differences were observed for the levels of LAB cocci, enterococci, pseudomonads, CPS and members of the Bacillaceae family between the powders of *T. molitor* and *A. domesticus*. Yeasts and moulds were undetectable for both matrices, while only mealworm powder showed pseudomonads below the detection level. The levels of TMM in both powders were a little lower than 106 CFU/g. The highest levels in mealworm powder were reached by LAB cocci (5.95 log CFU/g) followed by CPS, members of Bacillaceae family and LAB rods, which were all at cell densities above 105 CFU/g, while house cricket powder showed only LAB cocci at these levels. In general, *A. domesticus* powder showed lower levels of all microbial groups than *T. molitor* powder except the members of *Enterobacteriaceae* family.

The microbiology of insect powders soon after production has been studied by other authors considering different microbial groups. Bußler et al. [14] evaluated the total viable counts (on PCA) of *T. molitor* powder and observed levels of 7.72 log CFU/g of dry matter. Klunder et al. [11] analyzed the microbial loads of *T. molitor* powder after the insect were subjected to boiling and crushing reporting levels of 4.8 log CFU/g. The same authors also analyzed house crickets after boiling and stir-frying showing levels of 2.7 log CFU/g on PCA. In the same study, the members of *Enterobacteriaceae* family were 2.6 log CFU/g for mealworm and below the detection level in house cricket powder, while bacterial endospores were detected only in *A. domesticus* powder and counted at 1.5 log CFU/g. LAB were also the object of investigation by Klunder et al. [11], but their levels (ranging from 7.9 to 8.9 log CFU/g) were evaluated only after fermentation of the mixture mealworm powder/water at 30 °C. Information about the microbiology of *T. molitor* and *A. domesticus* are also available for the entire insects after freeze-drying [15]. Furthermore, Osimani et al. [46] also investigated on the hygiene of these insects reared under controlled conditions.

Only the study of Klunder et al. [11] considered the microbiological changes occurring during the refrigeration (4 °C) and ambient temperature (25 °C) storage of *A. domesticus* powder, but the monitoring period lasted 16 days. Thus, due to the different preparation and storage duration/conditions and the samples analyzed, a real comparison of data with those available in the literature is difficult. In general, the levels of TMM and enterobacteria registered for *A. domesticus* and *T. molitor* powders after 12 months of storage were comparable to those reported for the insects raised in the open field [11].

### 3.2. Culture-Independent Microbiological Analysis

**Figure 2:**
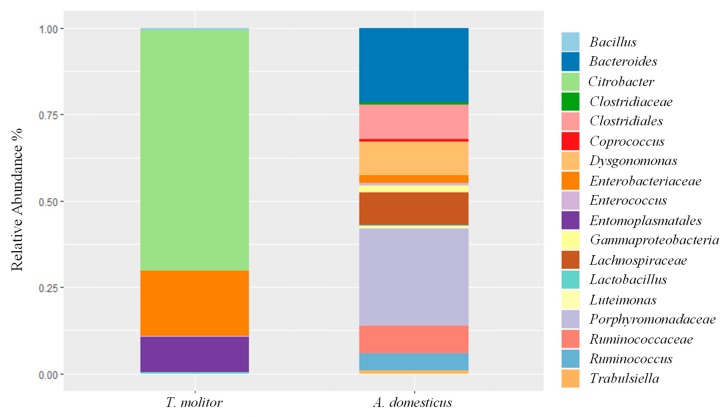
Relative abundances (%) of bacterial groups identified by MySeq Illumina in insect powders. Only taxonomic groups with at least two representative sequences per taxonomic unit were retained.

After processing of the demultiplexed FASTQ files using DADA2 package, we obtained 69,818 and 133,305 reads for house cricket and mealworm powder, respectively. Only taxonomic groups with, at least, two representative sequences per taxonomic unit were retained and the relative abundances were reported in Figure 2.

The diversity evaluation done using MiSeq Illumina identified members of the *Citrobacter* genus as the major components of the mealworm powder, which are commonly associated with the mid-gut of Lepidoptera insects [47], followed by *Enterobacteriaceae*. Members of *Porphyromonadaceae* family constituted the major bacterial group of house cricket powder. Many species of the family *Porphyromonadaceae* are part of the indigenous microbiota of the human and animal gastrointestinal tract and oral cavity [48]. To retrieve information at the species level, the most representative sequences of the two samples were manually blasted against the NCBI database. All *Enterobacteriaceae* OTUs were identified as belonging to *Salmonella enterica*/*Pseudocitrobacter faecalis*/*Cronobacter sakazakii*, while *Porphyromonadaceae* OTUs belonged to uncultured bacteria. No significant differences were found between observed and predicted (Chao1estimator) OTUs. Therefore, it is possible to capture the majority of OTUs present in each sample. Statistical analysis using the Kruskal-Wallis test revealed significant differences among *A. domesticus* and *T. molitor* (*p* < 0.05). This analysis showed a highest biodiversity in terms of bacterial species for *A. domesticus* powder. Regarding the lactic acid bacteria group, Illumina identified *Enterococcus* and *Lactobacillus* genera, generally found in entire mealworms [46].

Even though NGS analysis, as performed in this study, was based on DNA and this approach does not provide any indication on the viability of the detected species some safety issues arose from the composition of the microbiotas of the two insect powders analysed. In particular, the major groups belonged to enteric bacteria that are commonly found in several raw materials, such as *Enterobacteriaceae* family members in raw milk [49], meat [50] and vegetables [51]. Thus, considering insect powders as raw materials to be added as ingredient in food matrix formulations rather than as foods themselves, no particular food safety alerts concerning the major foodborne pathogens were shown by this study, even though the presence of *Salmonella* spp. deserves deepen investigations. However, several enterococci were isolated in viable form. Due to the antibiotic resistance gene transfer that could occur in some *Enterococcus* strains [52], a more comprehensive investigation on the isolates of this study should be done once insects flours will be used in food and feed formulations.

### 3.3. Characterization of LAB

**Figure 3:**
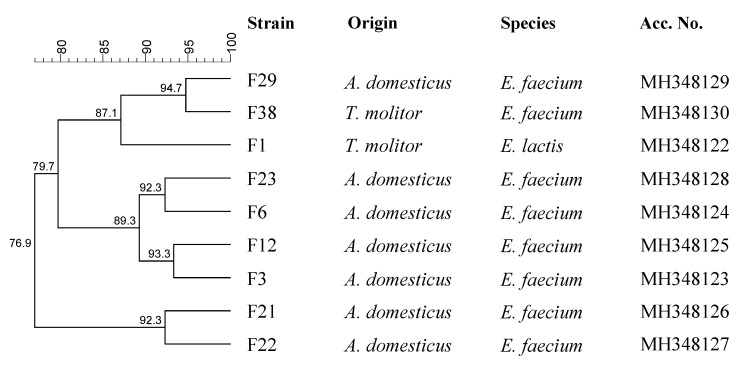
Dendrogram obtained with combined random amplification of polymorphic DNA (RAPD)-PCR patterns of *Enterococcus* strains.

The colonies grown on the media (MRS, M17 and KAA) specifically used for mealworm and house cricket powder LAB enumeration were collected and characterized. Barely 40 cultures were still considered presumptive LAB after Gram determination and catalase test. The microscopic investigation showed a coccus shape for all bacteria even though MRS allows the growth of rod LAB. The genetic typing showed nine different RAPD profiles, which corresponded to nine distinct strains (Figure 3). The sequencing of rRNA genes allotted all strains into *Enterococcus* genus.

In particular, *Enterococcus faecium* were identified from *A. domesticus* powder, while *E. faecium* and *Enterococcus lactis* were observed in mealworm powder as confirmed by species-specific multiplex PCR.

Although some information is available on the presence of LAB in edible insects [46], this is the first study aimed at increasinging the characterization of insect powder LAB. Mealworm larvae and their frass analyzed for the presence of LAB by culture-independent tools were found to host *Lactobacillus, Pediococcus*, and *Leuconostocaceae* when the investigation was done through Illumina, while *Lactococcus* spp., *Enterococcus* spp. and *Lactobacillus* spp. when denaturing gradient gel electrophoresis was used [17], but no information on their viability was reported. As anticipated above, Klunder et al. [11] investigated LAB during the fermentation of mealworm powder, starting from a batch prepared mixing the insect powder with water at a ratio of 40:60, which was subjected to 5 fermentation cycles using 10% inoculums from the previous cycle. LAB increased in time, but the acidifying species were not identified.

It was seen that the dominant LAB of *T. molitor* and *A. domesticus* powders were all members of *Enterococcus* genus. Enterococci are bacteria of intestinal origin often associated with food matrices, but they rarely represent starter cultures for the fermentation processes [53]. The presence of *Enterococcus* in food products is a direct consequence of faecal contaminations [53]. In the case of insects, they are transferred from their intestinal tracts. In general, the enterococci from insect powder did not show dangerous risk factors since all of them were not virulent and were sensitive to the 12 antibiotics commonly used for the treatment of human and animal infections.

### 3.4. Proximate Composition, Energy and Fatty Acid Profile of Insect Powders

The proximate composition of insect powders is summarized in Table 1.

The lipid value is 26.17% (Table 1) in *T. molitor* and 21.66% in *A. domesticus*. As expected, both insect species showed a high proportion of protein, 52.95% for *T. molitor* and 63.62% for *A. domesticus*. Insects contain chitin, a primary component of the exoskeleton of arthropods [54]; our analyses showed a chitin percentage of 14.42 for *T. molitor* and 5.50 for *A. domesticus*. Regarding moisture and ash, the values were between 3 and 5% for *T. molitor* and *A. domesticus*, respectively. The energy content (Table 1) varied slightly between the species, ranging from 1868.59 ± 2.26 kJ/100g for *T. molitor* to 1882.88 ± 3.65) kJ/100 g for *A. domesticus*. These results are comparable to the energy supplied by beef (1735 kJ/100 g) or fish (1662 kJ/100 g) [55]. The results of the proximate composition were comparable to those reported in literature [18,54,55,56] and put in evidence the high potential of insects as alternative sources of new and renewable animal proteins and fat [54,57].

Table 1 shows the fatty acid composition of edible insect’s powder. The amount of saturated fatty acids (SFA) ranged from 23.65% for *T. molitor* to 39.68% for *A. domesticus*. The two main components of the SFA were palmitic acid (C16:0) and stearic acid (C18:0). The highest amount of these fatty acids was observed in *A. domesticus*. Values detected in *T. molitor* powder were in agreement to those reported from Zielińska et al. [55] that showed 18% of palmitic acid and 3.8% of stearic acid in the larvae of *T. molitor*. The content of monounsaturated fatty acids (MUFA) varied from 24.66% in *A. domesticus* to 48.06% in *T. molitor*. The major MUFA of edible insect’s powder is oleic acid (C18:1 *n*-9). The highest content of oleic acid was observed in *T. molitor*. The fraction of polyunsaturated acids (PUFA) ranged from 28.28% in *T. molitor* to 35.66% (Table 1) in *A. domesticus*. Yang et al. [58] obtained similar values for crickets (33.8%). In particular, in this study, in *T. molitor* powder, the omega-6 (*n*-6) PUFA were higher than omega-3 (*n*-3) (2.3%). For the n-6 class, the most abundant FA was linoleic acid. Regarding *A. domesticus* powder, n-6 PUFA were higher than omega-3 (*n*-3). Linoleic acid was the most abundant fatty acid of the n-6 class similar to values observed by Yang et al. [58] in ground crickets (32.2%) and by Osimani et al. [18] in the same species. Furthermore, various data regarding the body composition of insects showed the variable composition of FA between species, origin and developmental stages [56].

### 3.5. Enzymatic Hydrolysis

**Figure 4:**
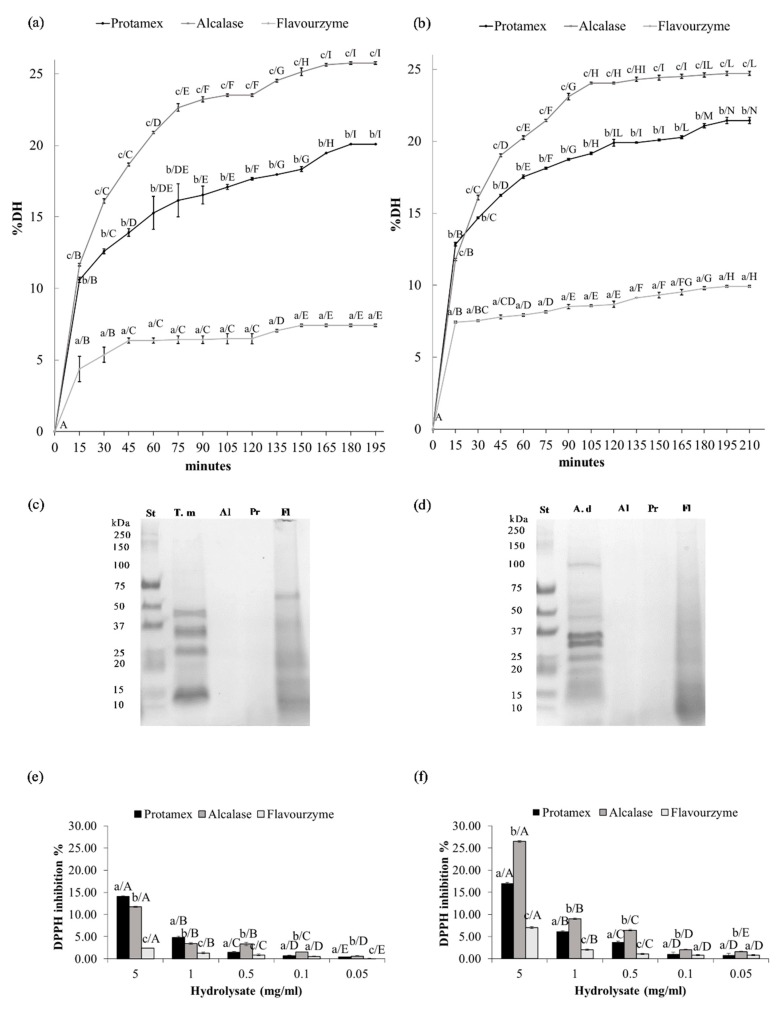
Physico-chemical properties: (**a**) Degree of hydrolysis of *T. molitor* powder; (**b**) Degree of hydrolysis of *A. domesticus* powder, different lowercase letters indicate significant differences between different enzymes (a, b, c...: *p* < 0.05), different uppercase letters indicate significant differences between different time points for each enzyme (A; B; C…*p* < 0.05); (**c**) SDS-PAGE of the total proteins (TM) and proteins hydrolisates, obtained with the enzymes alcalase AL, protamex Pr and flavourzyme Fl, from *T. molitor* powder at the end of the enzymatic process; (**d**) SDS-PAGE of the total proteins (TM) and proteins hydrolisates, obtained with the enzymes alcalase AL, protamex Pr and flavourzyme Fl, from *A. domesticus* powder at the end of the enzymatic process; (**e**) DPPH radical scavenging activity of protein hydrolysates of *T. molitor* powder; (**f**) DPPH radical scavenging activity of protein hydrolysates of *A. domesticus* powder. Different lowercase letters indicate significant differences between different enzymes at the same concentration (a, b, c...: *p* < 0.05), different uppercase letters indicate significant differences between different concentrations tested for each enzyme (A; B; C… *p* < 0.05); values are mean (three replications) ± standard deviations.

The enzyme selection is the most important factor in protein hydrolysis affecting the yield and physico-chemical properties of the final product. From the comparison of the three types of commercial proteases used, Alcalase from *Bacillus licheniformis* had provided the best results in hydrolyzing the total proteins of the *T. molitor* powder (Figure 4a) in terms of DH %, with increasing trend, followed by Protamex and Flavourzyme, which instead showed rather stable values over time (*p* < 0.05). The maximum DH% (between 20.1% of the enzyme Protamex and 25.8% of the enzyme Alcalase) were reached after 180 min of reaction.

The enzymatic reaction of hydrolysis to the powder of *A. domesticus* (Figure 4b) was better in terms of DH%, also in this case, with the enzyme Alcalase (*p* < 0.05). The plateau phase was reached at 195 min, where the maximum value of 24.6% (DH) (Figure 4d) was observed, which remains unchanged in subsequent measurements. Several authors had reported that, compared to other proteolytic enzymes, Alcalase allows superior protein recovery and provide hydrolysates with good functional properties [59,60,61,62,63]. Generally, alkaline proteases, including Alcalase, exhibit greater proteolytic activity than acid or neutral proteases such as Flavourzyme [62]. Yang et al. [64] reported that, among the proteases used, Alcalase had higher DH during the hydrolysis period, which suggested that Alcalase is more efficient than the other enzymes for preparing protein hydrolysates from edible insects. Tang et al. [23] obtained better results in terms of DH%, in *T. molitor* larvae, with a combination of Alcalase and Flavourzyme followed by slightly lower values obtained only with the enzyme Alcalase. Mizani et al. [60] reported that Alcalase, when used in combination with sodium sulphite and triton x-100, increased the yield of protein hydrolysates from *Penaeus semisulcatus* shrimp waste (heads) from 45.1% to 62–65%. This is related to a reduction of disulphide bonds and increased the solubility of proteins, as has been previously demonstrated in the case of soy products [65]. Recently, Alcalase has been used extensively in the hydrolysis of plant and animal proteins [20,21,66].

### 3.6. SDS PAGE

The SDS-PAGE electrophoresis (Figure 4c,d) showed that the electrophoretic pattern of the protein hydrolysates with respect to the total proteins extracted from the whole insect meals (T. m. and A. d. in Figure 4c,d), had a progressive loss of the bands at higher molecular mass, attesting to the efficiency of the reaction. According to the low DH obtained, the electrophoretic profile of Flavourzyme hydrolysates showed protein bands with a higher relative molecular mass than the other two enzymes, comparable to the profile of the total proteins of the whole samples (T. m. and A. d. in Figure 4c,d). In the study done by Kristinsson and Rasco [66], peptides with a lower molecular weight were observed with a higher DH. The enzyme Alcalase gave peptides at 65 kDa, while with Flavourzyme most of the peptides present a molecular mass of 70 kDa. At a higher degree of hydrolysis the bands with greater mass begin to disappear; this confirmed the fact that the molecular weight of the peptides formed by hydrolysis is associated with the degree of hydrolysis.

### 3.7. DPPH Radical Scavenging Activity

The DPPH assay showed that the protein hydrolysate of *T. molitor* powder (Figure 4e) had antioxidant properties, attested by inhibition of the DPPH radical up to 14.0% (Figure 4e). In particular, the hydrolysate obtained from the enzyme Protamex showed a higher antioxidant power in respect to the hydrolysate obtained with Alcalase and Flavourzyme only at the highest concentrations tested (5 and 1 mg/mL) (Figure 4e) (*p* < 0.05).

The DPPH assay on *A. domesticus* powder hydrolysate (Figure 4f) showed that the best antioxidant properties, resulting in an inhibition of DPPH radical up to 26.5%, were observed in the hydrolysates obtained using the enzyme Alcalase (*p* < 0.05). In Figure 4f it is evident a linear and dose-dependent relationship between the scavenging properties of the DPPH radical and the various concentrations tested, the linearity has been tested through the linear regression test obtaining the following values of R^2^: *A. domesticus* Alcalase 0.982 (*p* > 0.001); *A. domesticus* Protamex 0.976 (*p* > 0.001); *A. domesticus* Flavourzyme 0.994 (*p* > 0.001). These results were comparable to previous reports [23,64].

Similar results have already been reported in the literature, confirming that the degree of hydrolysis can significantly influence the antioxidant activity of the resulting hydrolysates, probably due to the presence of a high amount of low molecular weight active peptides [67,68,69]. Ahn et al., [70] stated that the molecular weight of peptides was related to their functional properties, with greater efficacy in bioactive peptides at a molecular weight of about 1.0–3.0 kDa. Taheri et al., [71] showed that protein hydrolysate fractions between 1.0 and 10 kDa had higher antioxidant power than higher molecular weight fractions. Therefore, since high DH means that more peptide bonds were cleaved, the protein would release lower molecular weight peptides to the hydrolysates, endowing the hydrolysates with high antioxidant activity, indicating that a certain degree of DH is necessary to the physico-chemical activity of hydrolysates. Our future analyses will be directed towards the specific amino-acid analysis of the hydrolysates obtained.

## 4. Conclusions

Insect powders have been subjected to a deep microbiological characterization in view of their application in wheat powder fermentations to obtain fortified products. The fortification of traditional food products represents a successful strategy to provide the necessary nutrients without substantial modification of the alimentary habits [72]. Generally, insects were found to be highly nutritious and to represent good sources of protein, fat, minerals, vitamins, and energy [54]. They are traditionally used as a food source in different countries, but nowadays, they are becoming globally increasingly attractive as a protein and fat source for humans and many types of pet and farm animals [73]. Insects are useful not only for their nutritional composition [74] but also for the transfer of other indispensable nutrients and micronutrients to the recipients [75]. Insect protein is readily available with protein quality values similar to, or slightly higher than, fish meat or soybean powder [74]. Moreover, proteins analyzed showed to be a suitable source of biologically active peptides to generate multifunctional hydrolysates that could be incorporated into functional foods or used as nutraceuticals or as natural alternatives to synthetic antioxidants. Further study is also needed in the characterization of edible insect peptides, optimization of functional properties, sensory evaluation, and establishing applications of these hydrolysates in food formulations [20,21].

## Figures and Tables

**Table 1:** Proximate composition (%), chitin (%), energy content (kJ/100g) and fatty acid composition (%) of the insect powders. Values are mean (three replications) ± standard deviation.

Proximate Composition	*Tenebrio molitor* (*T. molitor*)	*Acheta domesticus* (*A. domesticus*)
Moisture	3.12 ± 0.39	4.72 ± 0.15
Ash	3.32 ± 0.04	4.49 ± 0.04
Lipid	26.17 ± 0.21	21.66 ± 0.13
Protein	52.95 ± 0.33	63.62 ± 0.5
Chitin	14.42 ± 0.33	5.50 ± 0.5
Energy	1868.59 ± 2.26	1882.88 ± 3.65
Fatty acids		
Myristic acid (14:0)	2.35 ± 0.07	1.66 ± 0.07
Palmitic acid (16:0)	17.96 ± 0.14	25.56 ± 0.44
Palmitoleic acid (16:1 *n*-7)	1.67 ± 0.01	0.81 ± 0.02
9,12-Hexadecadienoic acid (16:2 *n*-4)	0.02 ± 0.01	0.08 ± 0.00
6,9,12-Hexadecatrienoic acid (16:3 *n*-4)	0.15 ± 0.00	0.11 ± 0.00
Stearic acid (18:0)	3.34 ± 0.05	12.47 ± 0.10
Oleic acid (18:1 *n*-9)	45.75 ± 0.38	22.59 ± 0.04
Vaccenic acid (18:1 *n*-7)	0.50 ± 0.66	0.93 ± 0.02
Linoleic acid (18:2 *n*-6)	25.73 ± 0.18	32.35 ± 0.42
γ-linolenic acid (18:3 *n*-6)	n.d.	n.d.
8,11,14-Octadecatrienoic acid (18:3 *n*-4)	0.01 ± 0.02	n.d.
α-Linolenic acid (18:3 *n*-3)	2.30 ± 0.03	1.75 ± 0.01
Stearidonic acid (18:4 *n*-3)	n.d.	0.04 ± 0.04
Eicosenoic acid (20:1 *n*-9)	0.13 ± 0.04	0.21 ± 0.00
Arachidonic acid (20:4 *n*-6)	n.d.	0.14 ± 0.01
Eicosatetraenoic acid (20:4, *n*-3)	n.d.	n.d.
Eicosapentaenoic acid (20:5, *n*-3)	0.01 ± 0.01	0.48 ± 0.04
Cetoleic acid (22:1 *n*-11)	0.01 ± 0.02	0.11 ± 0.00
Erucic acid (22:1 *n*-9)	0.01 ± 0.02	0.01 ± 0.00
Adrenic acid (22:4 *n*-6)	0.03 ± 0.05	n.d.
Osbond acid (22:5 *n*-6)	0.00 ± 0.01	n.d.
Docosapentaenoic acid (22:5 *n*-3)	n.d.	0.10 ± 0.01
Docosahexaenoic acid (22:6 *n*-3)	0.02 ± 0.02	0.63 ± 0.02
Nervonic acid (24:1 *n*-9)	2.35 ± 0.07	0.00 ± 0.00
Saturated	23.65 ± 0.12	39.68 ± 0.41
Monounsaturated	48.06 ± 0.20	24.66 ± 0.00
Polyunsaturated	28.28 ± 0.31	35.66 ± 0.41
Total *n*-3	2.33 ± 0.07	2.99 ± 0.02
Total *n*-6	25.77 ± 0.24	32.49 ± 0.43

n.d. = not detected.
